# Effectiveness of Feed-Based Monovalent *Aeromonas* Vaccine in Farmed Carp

**DOI:** 10.3390/microorganisms13081903

**Published:** 2025-08-15

**Authors:** Nimra Mubeen, Farzana Abbas, Muhammad Hafeez-ur-Rehman, Margaret Crumlish, Haris Mahboob, Muhammad Akmal, Ayesha Sadiqa, Talha Mahboob Alam, Samama Jalil

**Affiliations:** 1 Department of Fisheries and Aquaculture, University of Veterinary and Animal Sciences, Lahore 54000, Pakistan; 2021-phd-1030@uvas.edu.pk (N.M.); mhafeezurehman@uvas.edu.pk (M.H.-u.-R.); muhammad.akmal@uvas.edu.pk (M.A.); somamajalil@gmail.com (S.J.); 2 Institute of Aquaculture, University of Stirling, Stirling FK9 4LA, UK; margaret.crumlish@stir.ac.uk; 3 Atta Ur Rehman School of Applied Biosciences, National University of Sciences and Technology, Islamabad 44000, Pakistan; harismahboob0@gmail.com; 4 Department of Biology, University of Okara, Okara 56300, Pakistan; ayeshasadiqa456@gmail.com; 5 Department of Computer Science, Norwegian University of Science and Technology, 7491 Trondheim, Norway

**Keywords:** *Aeromonas hydrophila*, agglutination antibody titer test, *Ctenopharyngodon idella*, feed-based vaccine, lysozyme activity

## Abstract

*Aeromonas hydrophila* (*A. hydrophila*) is responsible for causing abdominal dropsy, swimming abnormalities, skin ulcerations, and pale gills in fish. Vaccination is an essential strategy for disease prevention in aquaculture. This study evaluated the efficacy of an oral vaccine against *A. hydrophila* in *Ctenopharyngodon idella* (*C. idella*). The vaccine was formulated as feed-based monovalent pellets, incorporating or spraying formalin-killed *A. hydrophila* on/into commercial feed with 30% crude protein. Mineral and fish oils were used as adjuvants at 10% of the feed. Prior to the trial, the experimental feed groups were subjected to quality and safety tests. Grass carp fingerlings (20 ± 5 g) were divided into seven groups (*n* = 20 per group): sprayed vaccinated feed with fish oil (SVFF), incorporated vaccinated feed with fish oil (IVFF), sprayed vaccinated feed with mineral oil (SVFM), incorporated vaccinated feed with mineral oil (IVFM), sprayed vaccinated feed (SVF), incorporated vaccinated feed (IVF), and a control group. Feed was provided at 3% of body weight for 60 days. Immunomodulation was investigated through lysozyme activity, antibody titers, and immunoglobulin M (IgM). The IVFF group showed significantly enhanced immunity and growth performance, with an 87% protection rate, 13% mortality, and the highest relative percentage survival (83%) following intraperitoneal *A. hydrophila* (6.8 × 10^9^ CFU/mL) challenge. Histological analysis indicated minimal pathological changes in the IVFF group compared to controls. Fish oil as an adjuvant enhanced immunity without adverse health effects. Overall, this study demonstrated that feed-based monovalent vaccines effectively improve immune responses and provide protection against *A. hydrophila* in *C. idella.*

## 1. Introduction

Aquatic organism diseases seriously constrain sustainable aquaculture expansion and development. Approximately every three to five years, a previously unidentified pathogen that causes a novel and unknown disease will emerge in worldwide aquaculture, spread quickly, including across national borders, and may cause substantial losses in production [1]. Motile *Aeromonas* septicemia (MAS) remains the most common disease in freshwater fishes, including common carp (*Cyprinus carpio* L.), eels (*Anguilla* spp.), catfish (*Ictalurus punctatus*), goldfish (*Carassius auratus*), and tilapia (*Oreochromis niloticus*), leading to significant economic losses globally, amounting to millions of dollars [2,3,4]. The largest production fish species in the world, grass carp (*C. idella*), accounts for around 16% of all freshwater aquaculture. It is an important economic fish that is farmed widely in China and other Asian nations [5]. The rapid development of the grass carp industry has made MAS caused by *A. hydrophila* a more serious problem in recent years [5].

Antibiotic-medicated feed is a common practice for managing MAS and has been helpful in feeding infected fish [6]. Antibiotics are frequently used in the treatment of bacterial fish diseases. These medications are widely used in veterinary medicine to treat infectious bacterial diseases in aquaculture, such as vibriosis, streptococcosis, and MAS, in order to minimize losses in the aquaculture sector [7].

Some bacterial species undergo mutation in unfavorable conditions after using antibiotics to survive in new conditions [8]. Antibiotic usage not only increases the risk of occupational exposure to antibiotics in farmers, but its bioaccumulation and toxic actions on aquatic ecosystems and antibiotic residue in cultured animals may be transferred and accumulated in human bodies [9].

Vaccination is one of the alternatives that have been proposed to overcome disease-caused mortality and morbidity after the restriction of using antibiotics in aquaculture [10]. Vaccination has been recognized as a safer and more sustainable alternative for preventing bacterial infections in aquaculture systems [11,12]. Fish vaccination can be delivered by immersion, oral, and injection methods. For large-scale fish farming, the immersion and injection methods are not perfectly suitable because of the high cost, and more work is required. As a result, it is critical to develop an effective and practical vaccine administration strategy for fish of all sizes and life stages, but still eliciting local and specific immune responses that continue for a long time [13].

Various experimental vaccines for aquaculture have been evaluated in neighboring countries such as India [14,15], Bangladesh [16], Iran [17], and China [18]. However, there is a scarcity of the literature on the development or use of fish vaccines in Pakistan. It is crucial to highlight that *A. hydrophila*, the primary cause of fish dropsy disease in Pakistan, has developed antibiotic resistance to many drugs [19].

Feed-based vaccination offers numerous advantages, including practicality, minimal stress for fish, reduced labor costs, applicability across all fish sizes, and the potential for repeated booster administration throughout the growth period in both cage and pond systems [20]. Vaccines given orally through feed can stimulate both systemic and mucosal immune responses, protecting the fish from pathogens and reducing the infection outbreaks systemically [21].

In this study, a feed-based vaccine against *A. hydrophila* was developed for aquaculture fish species in Pakistan. In many developing countries, including Pakistan, the vaccine industry faces major economic and social constraints that hinder the development of injectable vaccines. This study aims to provide a more effective and cost-efficient vaccination method by developing an oral vaccine with the best adjuvants to enhance production rates at reduced costs.

## 2. Materials and Methods

### 2.1. Experimental Site

This experiment was carried out in the Fish Hatchery Complex, Department of Fisheries and Aquaculture and Medical Laboratory Technology (MLT), UVAS, Ravi Campus, Pattoki.

### 2.2. Bacterial Recovery

The vaccine was formulated from previously isolated and procured *A. hydrophila* (AH-24123), a naturally infecting strain from carps (deposited at NCBI GenBank under accession number—MT249822.1) belonging to UVAS, Lahore. The stocked *A. hydrophila* (AH-24123) was reconfirmed on the basis of specific morphological, physiological, and biochemical characteristics [22]. Sequences of *A. hydrophila*, amplified using 27F and 1492R 16S rRNA-specific primers, showed 99% similarity to the standard *A. hydrophila* sequence in NCBI [23].

### 2.3. Vaccine Preparation

The preserved bacterial stocks at −80 °C were revived by spreading 100 µL on the tryptic soy agar (TSA) plate (Sigma-Aldrich^®^, St. Gallen, Switzerland). A single colony subculture was picked and inoculated in 5 mL of tryptic soy broth (TSB) (Sigma-Aldrich^®^, Switzerland), then cultured at 30 °C in an incubator for 24 h [24]. This culture was used for the enrichment of bacteria and inoculated into two 500 mL flasks, each having broth, and incubated at 30 °C for 24 h with an orbital shaker at 150 rpm. Bacterial cells from 24 h cultures in TSB were harvested by centrifugation at 6000× *g* for 15 min. The cells were washed 2–3 times with 0.9% (*w*/*v*) sterile saline, and the optical density (OD600) was adjusted to 0.67 to achieve a final bacterial concentration of 6.8 × 10^9^ CFU/mL. Serial dilution and bacterial colony counts were used to calculate the bacterial concentration after centrifugation, following the standard method [25]. Bacteria were inactivated by adding formalin (1% *v*/*v*) and kept at 30 °C for 4–6 h. Subsequently, the formalin-inactivated bacteria were centrifuged again at 6000× *g* for 15 min and washed 2–3 times with sterile saline solution to eliminate residual formalin. The sterility of the inactivated bacteria was assessed using an in vitro method to ensure complete bacterial inactivation [22]. Fish oil (Nutrifactor Laboratories (Pvt) Limited, Faisalabad, Pakistan) and mineral oil (Montanide ISA 201 VG (W/O/W) emulsion) (Acros Organics, A Thermo Fisher Scientific Brand, Waltham, MA, USA) were added at a concentration of 10% as adjuvants.

### 2.4. Development of Vaccinated Feed

Commercial powder feed with 30% crude protein content was purchased from Hi-Tech Feeds Mill (Pvt) Ltd. of Lahore, Pakistan, and divided into two portions for experimentation. In the first portion, vaccine was added via a spray method, while the second portion while the second portion used the vaccine incorporation method. The vaccine was added at a ratio of 1 L per 1 kg of commercial powder feed, ensuring a final concentration of 6.8 × 10^9^ bacterial cells per gram of fish feed. The monovalent vaccination was evenly distributed and appropriately incorporated into the fish feed powder using a homogenizer. As a control group, only sterile saline was added to the fish feed of the unvaccinated group. Prior to the feed-based immunization, the vaccine-added feed paste was finally loaded into the mini pellet machine to make a pellet and kept overnight at 30 °C [25]. Experimental treatments were designed based on the use of adjuvants (mineral and fish oil).

### 2.5. Fish Sampling

A total of 300 healthy *C. idella* fish with an average weight of 20 ± 5 g were purchased from the Fish Farm at UVAS, Ravi Campus, Pattoki. The fish were held and acclimated in an aquarium facility at the Fish Hatchery Complex, UVAS, for one week. Fish were fed daily on commercial powder feed. For the experimental trial, the fish were randomly allocated into 7 experimental groups of 14 tanks, each containing 20 fish, with two replicates. The experimental groups included (i) sprayed vaccinated feed with fish oil (SVFF), (ii) incorporated vaccinated feed with fish oil (IVFF), (iii) sprayed vaccinated feed with mineral oil (SVFM), (iv) incorporated vaccinated feed with mineral oil (IVFM), (v) sprayed vaccinated feed (SVF), (vi) incorporated vaccinated feed (IVF), and (vii) a control group (Table 1). Careful transfer of the fish into the tanks was ensured. Throughout the 60-day trial period, the fish were fed twice daily at 09:00 AM and 03:00 PM, with a feed rate equivalent to 3% of their body weight.

### 2.6. Quality Tests of Vaccinated Feed

Prior to initiating any in vivo fish studies, quality tests of vaccinated feed were performed. Proximate analysis, including estimation of crude protein (%), crude lipid (%), moisture (%), and ash (%), was performed according to the methods outlined in [26]. Additionally, palatability assessment [27] and evaluations of safety and water stability of the pelleted test diets were carried out [28].

### 2.7. Growth Parameters

During this study, various fish growth parameters were monitored to assess the impact of the experimental feed groups on the fish. These parameters included net weight gain (NWG), specific growth rate (SGR), and feed conversion ratio (FCR), as documented in Sughra et al. [22].

Net weight gain = average final weight − average initial weight

SGR = In (final body weight) − In (initial body weight) ÷ total days × 100

FCR = feed intake (g) ÷ net weight gain (g)

### 2.8. Serum Collection

Ten fish from each treatment group were selected for blood collection, with two fish sampled at a time. These blood samples were centrifuged at 6000 rpm for 20 min. Supernatants were transferred to centrifuged tubes and stored at 4 °C for further use [29].

### 2.9. Agglutination Antibody Titer Test

The serum agglutinating titer was assessed using a 96-well microtiter plate with round-bottom wells. The assay began with an initial 1:1 dilution (50 µL of phosphate-buffered saline [PBS]: 50 µL of serum), followed by two-fold serial dilutions of the serum. This was achieved by transferring 50 µL of the diluted serum into subsequent wells, each containing 50 µL of PBS, resulting in serum dilutions of 1:2, 1:4, 1:8, 1:16, 1:32, 1:64, 1:128, 1:256, 1:512, 1:1024, and 1:2048. Subsequently, 50 µL of inactivated *A. hydrophila* suspension (6.8 × 10^9^ CFU/mL) was added to each well. The microtiter plate was incubated at room temperature for 16–18 h. The endpoint of agglutination was defined as the highest serum dilution at which visible agglutination was observed, compared to a positive control. Agglutination antibody titers were expressed as the log2(x + 1) of the reciprocal of the highest serum dilution displaying visible agglutination. A negative control was included in the last well, containing only 50 µL of PBS without serum or bacterial suspension [30].

### 2.10. Serum Lysozyme Activity

Sterile *Staphylococcus aureus* (USA-05 Uropathogen *S. aureus*) was added to a phosphate buffer solution (2.25 g Na_2_HPO_4_, 10.75 g NaHPO_4_, and 125 mL distilled water). The absorbance of the bacterial suspension was adjusted to an optical density (OD450) of 0.6–0.7 using a spectrophotometer (U2020, IRMECO, Lütjensee, Germany). A serum sample (1 mL) was mixed with a bacterial suspension (2 mL) and incubated five times at one-minute intervals in a water bath at 40 °C. The absorbance at 450 nm was measured [31].

Units of enzyme activity per 1 mL of serum were calculated using the formula below.

Units/mL = (ΔA450/min − ΔA450/min) (df) ÷ (0.001) (0.01)

### 2.11. Total IgM Contents and Total Protein

Total protein was determined by the Bradford test, and IgM was calculated following the methods described in Anderson and Siwicki [32]. Briefly, fish serum (100 µL) and an equal volume of 12% polyethylene glycol were mixed. The sample was kept in an incubator at 37 °C for 2 h. After that, the sample was centrifuged at 1500 rpm for 10 min. When calculating the total IgM content, the protein content of the supernatant was subtracted from the protein content of the plasma.

### 2.12. Challenge Test

Fifteen fish from each treatment and control group were challenged with 0.1 mL of a bacterial dosage (6.8 × 10^9^ CFU/mL) as documented by Monir et al. [25]. The relative percentage survival (RPS) was calculated 14 days after the vaccination trial [33].

RPS = 1 − [(vaccinated fish mortality % age)/(control fish mortality % age)] × 100

### 2.13. Histopathology

Fish liver and intestine samples (2 fish from each treatment and control group) were processed at the Pathology Laboratory of UVAS to examine histopathological alterations after the challenge study. Histological analysis was performed following the protocol outlined by Yang et al. [34]. This involved careful preparation of tissue samples and subsequent microscopic examination to evaluate any structural changes or abnormalities in the liver and intestinal tissues of the fish specimens.

### 2.14. Statistical Analysis

All experimental data were expressed as mean values ± standard deviation (mean ± SD). Prior to statistical analysis, the normality of the data was evaluated using the Shapiro-Wilk test, and the homogeneity of variances was assessed using Levene’s test. A one-way analysis of variance (ANOVA) based on a completely randomized design (CRD) was conducted to determine statistically significant differences among the treatment groups. When significant effects were observed, Duncan’s Multiple Range Test (DMRT) was applied for post hoc pairwise comparisons [25]. All statistical analyses were performed using SPSS version 20.0, with the significance level set at *p* < 0.05 [35].

## 3. Results

### 3.1. Vaccinated Feed Quality Tests

A feed stability test was performed to assess the stability of the vaccine-incorporated feed, revealing significantly higher *p* < 0.05 values in the vaccinated groups (80.9 ± 0.95%) compared to the control group (70.0 ± 2.82%). Additionally, the feed palatability test showed significantly higher values compared to the control. Notably, group IVFF exhibited particularly high feed palatability (0.73 ± 0.17) compared to the control (0.50 ± 0.14) (Table 2).

A safety test was conducted on both the feed and the fish after feeding the vaccinated feed for one week. Vaccinated feed samples and the swabs were collected from the fish mouth, intestines, and gill, then swabbed on plates, and incubated at 30 °C for 24 h to assess bacterial growth. After incubation, no bacterial growth was observed on the TSA plates, indicating the absence of bacterial contamination in both the feed and the fish samples. This comprehensive assessment ensured the safety and quality of the feed and the well-being of the experimental fish throughout this study. This approach confirms bacterial identity, develops a stable and palatable vaccine, and conducts rigorous feed-safety testing. The results show the vaccine is safe for use in aquaculture, in addition to being effective in eliciting an immune response.

### 3.2. Proximate Analysis of Vaccinated and Control Feed

The proximate analysis of the feed showed significant differences for crude protein (%) and crude lipid (%) in all vaccinated feed groups compared to the control feed, but moisture (%) and ash (%) showed no significant difference. Among the vaccinated feed groups, group IVFF exhibited the crude protein (33.2 ± 3.88%), crude lipid (6.9 ± 0.04%), and moisture (10.4 ± 0.56%) values compared to the control group’s crude protein (30.0 ± 2.82%), crude lipid (4.5 ± 0.14%) (Table 3).

### 3.3. Proximate Analysis of Vaccinated and Control Fish

The proximate analysis of the fish showed significant differences between the vaccinated and control groups. Overall, all vaccinated groups exhibited notably higher protein values compared to the control group. Particularly, fish from group IVFF displayed the highest crude protein (*p* < 0.05) content among the vaccinated groups, indicating a robust response to vaccination. Significant differences in crude protein content (%) were observed among all dietary groups, ranked as follows: IVFF (26.1 ± 2.96) > SVFF (23.5 ± 0.92) > IVFM (21.2 ± 0.58) > SVFM (21.0 ± 2.56) > IVF (18.3 ± 2.38) > SVF (19.7 ± 3.29) > C (16.5 ± 0.42) (Table 4).

### 3.4. Growth Parameters of Fish

During the experimental trial, various growth parameters such as NWG, SGR, and FCR were recorded to evaluate the performance of fish growth. The results revealed that the group receiving incorporated vaccinated feed with fish oil (IVFF) exhibited numerically higher NWG (9.8 ± 0.82 g), SGR (0.85 ± 0.05%/day), and lower FCR (1.1 ± 0.14 g/g) (Table 5). However, in comparison to SVFM, IVFM, and SVFF, among other vaccinated groups, these changes were not statistically significant (*p* > 0.05). These findings reveal that while the IVFF treatment can potentially enhance growth performance, the observed trends were not consistently supported by significant differences in all growth parameters.

### 3.5. Agglutination Antibody Titer Test

Agglutination antibody titer tests were performed on days 1, 14, 28, 42, and 56. The mean antibody titer values showed a gradual increase from day 1 to day 56. Significantly higher antibody titer values (*p* < 0.05) were observed in the vaccinated feed groups compared to the control group. Moreover, there was a notable difference between the incorporated vaccinated feed groups and the sprayed vaccinated feed groups (0.38 ± 0.02), with the incorporated feed groups displaying higher antibody titer values (0.92 ± 0.07). Among the vaccinated feed groups, group IVFF, which received the incorporated vaccine with fish oil, exhibited the highest mean antibody titer value of (0.92 ± 0.07) compared to the control group (0.23 ± 0.01) (Table 6).

### 3.6. Serum Lysozyme Activity

Serum lysozyme activity was evaluated at specific intervals following the vaccinated feed trial, specifically on days 1, 14, 28, 42, and 56. Significant differences (*p* < 0.05) were observed among all dietary groups, with the lysozyme activity ranking as follows: IVFF > SVFF > IVFM > SVFM > IVF > SVF > C. The incorporated vaccinated feed group IVFF demonstrated the highest lysozyme activity (5960 ± 424.2 U/mL) among the experimental groups (3370 ± 436.9 U/mL) and the control group (2553 ± 77.7 U/mL) (Table 7).

### 3.7. Total IgM Contents

The total IgM contents were measured on days 1, 14, 28, 42, and 56. Fish from the incorporated vaccinated feed groups exhibited significantly higher values (*p* < 0.05) compared to those from the sprayed vaccinated feed groups. At day 56, vaccinated feed group IVFF showed the highest IgM (0.68 ± 0.07) contents among the vaccinated feed groups (0.36 ± 0.03) and the control group (0.21 ± 0.01) (Table 8).

### 3.8. Histopathology

**Figure 1:**
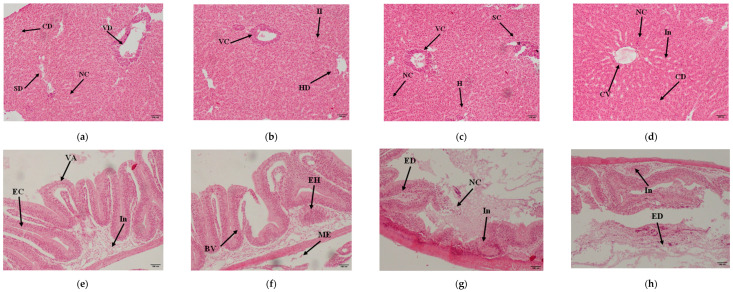
Histopathological changes in *C. idella*’s liver and intestine in vaccinated and control feed. (**a**) Fish liver of group control showing cellular disorganization (CD), necrosis (NC), sinosidal dilation (SD), and vacuolar degeneration (VD) (×40); (**b**) Fish liver of group SVF, showing hepatocyte degeneration (HD), vascular congestion (VC), and inflammatory infiltration (II) (×40); (**c**) Fish liver of group SVFM, showing vacuolar congestion (VC), necrosis (NC), sinusoidal consolidation (SC), and hemorrhage (H) (×40); (**d**) Fish liver of group IVF, showing cellular vacuolization (CV), necrosis (NC), cellular degeneration (CD), and inflammation (In) (×40); (**e**) Fish intestine of group control, showing villous atrophy (VA), inflammation (In), and epithelial congestion (EC) (×10); (**f**) Fish intestine of group SVF, showing epithelial hyperplasia (EH), mildly blunted villi (BV), and submucosal edema (ME) (×10); (**g**) Fish intestine of group SVFF showing epithelial degeneration (ED), inflammation (In), and necrosis (NC) (×10); (**h**) Fish intestine of group IVF, showing inflammation (In) and epithelial disruption (ED) (×10).

Notable changes in the *C. idella* liver and intestine were observed. Fish liver showed the fatty liver, apoptosis, granular deposition, necrosis, iso-organized hepatic cords, narrowed sinusoids, degenerated hepatocytes, diffuse inflammation, necrotic hepatic, and degenerated hepatocytes, and granular deposition. Fish intestine showed a reduction in the number of goblet cells, necrotic epithelial cells, enterocytes, mucosal epithelium, serosa presentation, hemorrhages, blood vessel congestion, necrosis, and epithelial necrosis within the lumen (Figure 1).

### 3.9. Challenge Test and Relative Percentage Survival Rate

Fish were monitored for signs of dropsy, fin hemorrhages, necrosis, and mortality for 14 days after the vaccination trial. The results revealed varying mortality rates across different dietary groups. In the incorporated feed group, IVFF demonstrated a significantly lower mortality rate of 13%, with complete protection observed against *A. hydrophila*. Vaccinated feed group IVFF exhibited a protection rate of 87%, a mortality rate of 13%, and a corresponding survival rate of 83%. Conversely, the control feed experienced the highest mortality rate of 80%. Relative survival rate analysis indicated that group IVFF (83%) provided better protection compared to the other vaccinated feed (18%), highlighting its efficacy in preventing *A. hydrophila* infections (Table 9).

After the challenge test, infected fish exhibited external signs including skin necrosis, hemorrhages on the dorsal and caudal fins, and scale detachment. Internal clinical symptoms included a pale liver and ruptured kidney. Isolation and bacteriological examination of tissue samples confirmed the presence of *Aeromonas hydrophila*, thereby establishing the diagnosis.

## 4. Discussion

Pellet water stability is crucial for the manufacturing of aquaculture diets. For benthic fish species, such as sea bass and grouper, which require sinking and water-stable feed, pellet disintegration is a key consideration. Pellets with low water stability may disintegrate easily, leading to nutrient leaching before fish intake and potentially deteriorating water quality [28]. In this study, the vaccine was incorporated into the feed, which could potentially affect the feed’s water stability. However, no significant difference in water stability was observed between the vaccinated pellets and the commercial pellets, indicating that the vaccinated feed maintains water stability comparable to that of the commercial feed.

Palatability is crucial for effective aquaculture. Feed with low palatability can decrease fish feed intake, potentially hindering the delivery of essential nutrients or antigens to the gut. The vaccinated feed in this study exhibited high palatability compared to the other feed. Ayisi et al. [36] reported that palm oil enhances feed intake, which aligns with the observation that improved palatability leads to increased feed consumption and overall nutrient intake [37]. Conversely, poor feed palatability can reduce intake and contribute to growth retardation in fish [27]. The feed-based monovalent vaccine used in this study demonstrated high palatability (0.73 ± 0.17) and a significant (*p* < 0.05) improvement compared to the commercial pellet (0.50 ± 0.14) after 1 h of feeding. This suggests that the incorporation of fish oil in the feed-based monovalent vaccine did not adversely affect palatability or feed intake. Additionally, fish oil has been used in fish vaccines to enhance the immune response by improving antigen delivery and efficacy. This approach boosts both cell-mediated and humoral immunity, and the use of fish oil as an adjuvant is known to improve the overall effectiveness of vaccines in aquaculture. Calder [38] reported that fish oil increases endotoxin and autoimmunity model survival, reduces both acute and chronic inflammatory responses, and increases the survival of grafted organs. Clinically, fish oil supplements may help with both acute and chronic inflammatory diseases.

Mohamad et al. [39] demonstrated that commercially available feed was combined with a polyvalent vaccine to ensure that fish, already accustomed to the feed, would readily accept it. However, feed mixing and pelleting processes might affect the feed-based monovalent vaccine nutrient compositions. The nutrient compositions—protein, lipid, carbohydrate, and ash—remained unchanged. However, moisture content was significantly higher in the vaccinated pellets compared to the commercial feed. This increase in moisture is likely due to the additional moisture introduced when the vaccine was incorporated into the ground pellet before it was re-pelleted using an extruder. Despite the higher moisture content, it remains within an acceptable range. Excessive moisture in feed pellets can lead to mold growth and reduced shelf life [40]. In this research, there were no significant differences in moisture and ash content, whereas significant differences were observed in crude protein and crude lipid.

Oral vaccination had no adverse effects on fish growth or nutritional digestion [41,42]. Conversely, Fraser et al. [43] suggested that vaccination could potentially reduce fish growth due to increased metabolic rates from ongoing immune system stimulation. In contrast, Beck et al. [44] and Ismail et al. [45] demonstrated significantly improved growth performance following oral immunization, potentially due to adjuvant use at a concentration of 10% oil. This study observed an increase in net weight gain, indicating enhanced overall growth and development in fish receiving the vaccinated feed, highlighting the benefits of this approach in aquaculture.

Antibody levels are a major parameter for evaluating specific immune responses. Antibody levels continued to rise, peaking in the fourth week, indicating enhanced immunity and increased antibody production against bacterial diseases [46,47]. Purwanti and Suminto [48] suggested that an increase in leukocyte concentration positively impacts antibody production, thereby enhancing the body’s resistance to foreign invaders. Serum antibody levels in immunized brood catfish groups, vaccinated with a bivalent vaccine (*A. hydrophila* and *A. veronii*), were higher than in unvaccinated control groups. These findings are consistent with similar research conducted in Indonesia [49]. This study confirmed a significant increase in antibody levels (*p* < 0.05) in fish fed with the monovalent incorporated vaccinated feed (group IVFF) compared to the control group after immunization.

Lysozyme, a crucial component of innate immunity, particularly in bacterial defense, exhibited significantly increased activity in vaccinated fish [50,51]. Sirimanapong et al. [52] reported significantly higher lysozyme activity in vaccinated tilapia three weeks post-vaccination. In contrast, do Vale Pereira et al. [53] reported no significant difference in lysozyme activity between vaccinated and unvaccinated fish post-vaccination. The observed increase in immune response in vaccinated fish (group IVFF) may be correlated with the enhanced lysozyme activity.

Ismail et al. [13] reported the increase in serum IgM antibody levels to the effective transport of antigens from the polyvalent vaccine to the distal gut segment of fish, triggering both local and systemic immune responses. Firdaus-Nawi et al. [20] emphasized that the second gut segment of fish is crucial for oral vaccination as it facilitates antigen transport and presentation to intra-epithelial macrophages, which then migrate to lymphoid organs to initiate a systemic immune response [53]. These findings are consistent with those reported by Adelmann et al. [54], Firdaus-Nawi et al. [20], Chideroli et al. [55], Ismail et al. [45], and others, who also observed a significant increase in IgM antibody levels following oral vaccination. Furthermore, the serum IgM levels against the cross-protective *A. hydrophila* antigen were statistically higher (*p* < 0.05) in the IVFF group on the 56th day. This indicates that the IVFF vaccine group induced both humoral and mucosal cross-immune responses when challenged with the *A. hydrophila* antigen.

Song et al. [56] investigated intestinal swelling in grass carp (*C. idella*) due to infection by the *A. hydrophila* strain. Histological analysis of vaccinated fish revealed inflammatory cell infiltrations near the outer pancreas, polymorphic inflammatory cells near the outer spleen, and inflammatory cell infiltrations at the injection site and intestine. In the present study, fish from the control group exhibited deteriorative alterations in the liver, kidney, and gills, possibly due to naturally occurring *A. hydrophila* infection in the water, exacerbated by factors such as stress and changes in environmental conditions [22]. These findings align with a recent study from India, where histopathological examination of *Pangasianodon hypophthalmus* showed normal architecture of the gills, liver, and kidney after oral vaccination against *A. hydrophila*. In contrast, fish in the control group displayed several histopathological abnormalities in all examined organs [57]. The present study corroborates these results, showing significant protection in vaccinated fish compared to unvaccinated fish. Histological analysis revealed distinct changes in the liver and intestine of control fish. The liver showed signs of fatty liver, apoptosis, iso-organized hepatic cords, narrowed sinusoids, diffuse inflammation, necrotic hepatic cells, and granular deposition. The intestine showed a reduction in goblet cells, necrotic epithelial cells, enterocytes, mucosal epithelium, and serosa presentation, as well as hemorrhages, blood vessel congestion, and epithelial necrosis within the lumen. These histopathological changes underscore the immunological response triggered by vaccination, enhancing the fish’s ability to resist *A. hydrophila* infection.

Chettri et al. [58] reported that effective vaccines provide substantial protection and reduce mortality rates in fish. Similarly, Gravningen et al. [59] reported an 80% RPS, while Villumsen et al. [60] and Fredriksen et al. [61] reported RPS rates of 78% and 77.5%, respectively, post-vaccination. Improved survival rates among vaccinated fish are crucial for farmers as they directly correlate with better harvest outcomes. The current investigation revealed protection rates ranging from 40% to 87% in fish fed with vaccinated feed, compared to only 20% in the control group. The highest RPS, at 83%, was recorded in the group fed with incorporated vaccinated feed containing fish oil (IVFF), while the lowest, at 18%, was observed in the group with sprayed vaccinated feed. This high RPS indicates good vaccine efficacy.

The adoption of fish vaccination for disease prevention is gaining momentum in developing nations such as India and Bangladesh, but remains relatively unexplored in Pakistan’s aquaculture industry. The current study aims to address this gap by initiating the development of oral vaccines to enhance fish health management and disease prevention. Although the experimental inactivated vaccine showed promising results, challenges remain in antigen selection, recombinant production, cost-effectiveness, administrative feasibility, safety, and scalability. Further research and refinement are necessary to overcome these challenges and advance vaccine development in aquaculture.

## 5. Conclusions

Fish vaccination remains an underexplored area in Pakistan, with limited prior experimental or commercial application. This study provides initial evidence that feed-based oral vaccines incorporating formalin-killed *A. hydrophila* with fish oil enhance and stimulate immune responses in *C. idella* under controlled conditions. Among the tested methods, the incorporated vaccine delivery showed comparatively better outcomes in terms of stability, safety, and immune indicators than the spray method. However, these findings are preliminary and based on a limited-scale laboratory trial. Further validation through larger, long-term field studies is necessary to assess the broader applicability and reliability of this approach in aquaculture health management.

## Figures and Tables

**Table 1:** Treatment groups of sprayed and incorporated vaccinated feed.

	(Control)	T1	T2	T3
Sprayed vaccinated feed	Commercial feed	Sprayed vaccinated feed (SVF)	Sprayed vaccinated feed with mineral oil (SVFM)	Sprayed vaccinated feed with fish oil (SVFF)
Incorporated vaccinated feed		Incorporated vaccinated feed (IVF)	Incorporated vaccinated feed with mineral oil (IVFM)	Incorporated vaccinated feed with fish oil (IVFF)

**Table 2:** Feed stability and palatability test of vaccinated and control feed.

Treatments	Feed Stability (%)	Feed Palatability
C	70.0 ± 2.82 ^c^	0.50 ± 0.14 ^c^
SVF	72.3 ± 6.14 ^b^	0.51 ± 0.28 ^c^
IVF	73.2 ± 4.51 ^b^	0.53 ± 0.42 ^c^
SVFM	72.5 ± 1.61 ^b^	0.65 ± 0.07 ^b^
IVFM	74.2 ± 5.97 ^b^	0.61 ± 0.01 ^b^
SVFF	75.7 ± 8.01 ^b^	0.60 ± 0.28 ^b^
IVFF	80.9 ± 0.95 ^a^	0.73 ± 0.17 ^a^

C (control); SVF (sprayed vaccinated feed); IVF (incorporated vaccinated feed); SVFM (sprayed vaccinated feed with mineral oil); IVFM (incorporated vaccinated feed with mineral oil); SVFF (sprayed vaccinated feed with fish oil); IVFF (incorporated vaccinated feed with fish oil). Values are shown as mean ± SD (*n* = 2). Different lowercase letters within the same column indicate statistically significant differences (*p* < 0.05) based on post-hoc comparisons.

**Table 3:** Proximate analysis of vaccinated and control feed.

Treatments	Crude Protein (%)	Crude Lipid (%)	Ash (%)	Moisture (%)
C	30.0 ± 2.82 ^b^	4.5 ± 0.14 ^c^	8.5 ± 0.70 ^ab^	10.0 ± 2.82 ^b^
SVF	31.2 ± 0.25 ^ab^	3.4 ± 0.09 ^d^	8.3 ± 0.28 ^b^	10.3 ± 0.42 ^b^
IVF	31.5 ± 1.41 ^ab^	5.9 ± 0.05 ^ab^	8.8 ± 1.13 ^a^	12.2 ± 3.11 ^ab^
SVFM	32.0 ± 2.82 ^a^	5.5 ± 0.14 ^bc^	9.5 ± 0.05 ^a^	16.0 ± 1.41 ^a^
IVFM	32.4 ± 3.45 ^a^	6.7 ± 0.42 ^ab^	8.0 ± 1.41 ^c^	11.0 ± 2.82 ^ab^
SVFF	32.7 ± 5.30 ^a^	5.8 ± 1.13 ^ab^	8.5 ± 2.12 ^ab^	14.7 ± 0.14 ^ab^
IVFF	33.2 ± 3.88 ^a^	6.9 ± 0.04 ^a^	8.2 ± 0.28 ^b^	10.4 ± 0.56 ^b^

C (control); SVF (sprayed vaccinated feed); IVF (incorporated vaccinated feed); SVFM (sprayed vaccinated feed with mineral oil); IVFM (incorporated vaccinated feed with mineral oil); SVFF (sprayed vaccinated feed with fish oil); IVFF (incorporated vaccinated feed with fish oil). Values are shown as mean ± SD (*n* = 2). Different lowercase letters within the same column indicate statistically significant differences (*p* < 0.05) based on post-hoc comparisons.

**Table 4:** Proximate analysis of vaccinated and control fish.

Treatments	Crude Protein (%)	Crude Lipid (%)	Ash (%)	Moisture (%)
C	16.5 ± 0.42 ^c^	2.2 ± 0.74 ^ab^	1.2 ± 0.03 ^a^	77.0 ± 0.67 ^b^
SVF	19.7 ± 3.29 ^bc^	2.2 ± 0.11 ^ab^	1.1 ± 0.06 ^a^	79.0 ± 0.45 ^ab^
IVF	18.3 ± 2.38 ^bc^	1.8 ± 0.48 ^ab^	1.2 ± 0.02 ^a^	78.0 ± 1.07 ^b^
SVFM	21.0 ± 2.56 ^abc^	2.3 ± 0.31 ^ab^	1.2 ± 0.07 ^a^	76.4 ± 0.78 ^c^
IVFM	21.2 ± 0.58 ^abc^	1.3 ± 0.84 ^b^	1.1 ± 0.11 ^a^	78.6 ± 0.49 ^ab^
SVFF	23.5 ± 0.92 ^ab^	2.4 ± 0.87 ^ab^	1.2 ± 0.11 ^a^	77.6 ± 0.56 ^bc^
IVFF	26.1 ± 2.96 ^a^	2.7 ± 0.14 ^a^	1.1 ± 0.05 ^a^	80.2 ± 0.41 ^a^

C (control); SVF (sprayed vaccinated feed); IVF (incorporated vaccinated feed); SVFM (sprayed vaccinated feed with mineral oil); IVFM (incorporated vaccinated feed with mineral oil); SVFF (sprayed vaccinated feed with fish oil); IVFF (incorporated vaccinated feed with fish oil). Values are shown as mean ± SD (*n* = 2). Different lowercase letters within the same column indicate statistically significant differences (*p* < 0.05) based on post-hoc comparisons.

**Table 5:** Growth parameters of vaccinated and control fish.

Treatments	NWG (g)	SGR (%/day)	FCR (g/g)
C	4.9 ± 0.81 ^c^	0.59 ± 0.01 ^ab^	2.0 ± 0.71 ^a^
SVF	8.3 ± 0.43 ^ab^	0.64 ± 0.02 ^b^	1.2 ± 0.28 ^ab^
IVF	6.2 ± 0.35 ^bc^	0.72 ± 0.07 ^ab^	1.4 ± 0.42 ^ab^
SVFM	7.7 ± 0.86 ^abc^	0.72 ± 0.01 ^ab^	1.2 ± 0.28 ^ab^
IVFM	7.1 ± 2.89 ^abc^	0.88 ± 0.09 ^a^	1.0 ± 0.03 ^b^
SVFF	7.9 ± 0.09 ^abc^	0.80 ± 0.24 ^ab^	1.1 ± 0.01 ^b^
IVFF	9.8 ± 0.82 ^a^	0.85 ± 0.05 ^ab^	1.1 ± 0.14 ^ab^

C (control); SVF (sprayed vaccinated feed); IVF (incorporated vaccinated feed); SVFM (sprayed vaccinated feed with mineral oil); IVFM (incorporated vaccinated feed with mineral oil); SVFF (sprayed vaccinated feed with fish oil); IVFF (incorporated vaccinated feed with fish oil); NWG (net weight gain); SGR (specific growth rate); FCR (feed conversion ratio). Values are shown as mean ± SD (*n* = 2). Different lowercase letters within the same column indicate statistically significant differences (*p* < 0.05) based on post-hoc comparisons.

**Table 6:** Agglutination antibody test of vaccinated and control fish.

Treatments	Day 14	Day 28	Day 42	Day 56
C	0.24 ± 0.03 ^b^	0.23 ± 0.01 ^d^	0.23 ± 0.01 ^d^	0.23 ± 0.01 ^e^
SVF	0.27 ± 0.04 ^b^	0.28 ± 0.05 ^c^	0.34 ± 0.06 ^cd^	0.38 ± 0.02 ^d^
IVF	0.29 ± 0.01 ^b^	0.29 ± 0.04 ^c^	0.36 ± 0.04 ^cd^	0.39 ± 0.01 ^d^
SVFM	0.32 ± 0.03 ^ab^	0.35 ± 0.03 ^bc^	0.44 ± 0.06 ^bcd^	0.48 ± 0.06 ^cd^
IVFM	0.35 ± 0.04 ^ab^	0.41 ± 0.06 ^bc^	0.46 ± 0.08 ^bc^	0.54 ± 0.06 ^b^
SVFF	0.51 ± 0.14 ^ab^	0.58 ± 0.16 ^ab^	0.62 ± 0.11 ^b^	0.68 ± 0.06 ^b^
IVFF	0.62 ± 0.29 ^a^	0.76 ± 0.21 ^a^	0.82 ± 0.15 ^a^	0.92 ± 0.07 ^a^

C (control); SVF (sprayed vaccinated feed); IVF (incorporated vaccinated feed); SVFM (sprayed vaccinated feed with mineral oil); IVFM (incorporated vaccinated feed with mineral oil); SVFF (sprayed vaccinated feed with fish oil); IVFF (incorporated vaccinated feed with fish oil). Values are shown as mean ± SD (*n* = 2). Different lowercase letters within the same column indicate statistically significant differences (*p* < 0.05) based on post-hoc comparisons.

**Table 7:** Serum lysozyme activity of vaccinated and control fish.

Treatments	Day 14	Day 28	Day 42	Day 56
C	1850 ± 70.0 ^d^	2080 ± 58.6 ^e^	2269 ± 72.1 ^e^	2553 ± 77.7 ^d^
SVF	2110 ± 1.55 ^d^	2269 ± 72.1 ^e^	2660 ± 56.5 ^d^	3370 ± 436.9 ^cd^
IVF	1869 ± 98.2 ^d^	2265 ± 21.2 ^e^	2800 ± 14.1 ^d^	3300 ± 424.2 ^c^
SVFM	3220 ± 28.2 ^c^	3927 ± 68.5 ^d^	4280 ± 282.8 ^c^	4450 ± 282.8 ^b^
IVFM	4208 ± 130.1 ^b^	4186 ± 141.2 ^c^	4290 ± 282.8 ^c^	4350 ± 282.8 ^b^
SVFF	4349 ± 167.5 ^b^	4729 ± 60.8 ^b^	5067 ± 68.5 ^b^	5469 ± 67.8 ^a^
IVFF	4834 ± 84.1 ^a^	5170 ± 141.2 ^a^	5802 ± 96.8 ^a^	5960 ± 424.2 ^a^

C (control); SVF (sprayed vaccinated feed); IVF (incorporated vaccinated feed); SVFM (sprayed vaccinated feed with mineral oil); IVFM (incorporated vaccinated feed with mineral oil); SVFF (sprayed vaccinated feed with fish oil); IVFF (incorporated vaccinated feed with fish oil). Values are shown as mean ± SD (*n* = 2). Different lowercase letters within the same column indicate statistically significant differences (*p* < 0.05) based on post-hoc comparisons.

**Table 8:** IgM levels of vaccinated and control fish.

Treatments	Day 14	Day 28	Day 42	Day 56
C	0.20 ± 0.01 ^b^	0.20 ± 0.01 ^d^	0.21 ± 0.01 ^d^	0.21 ± 0.01 ^e^
SVF	0.22 ± 0.01 ^b^	0.27 ± 0.02 ^cd^	0.29 ± 0.01 ^c^	0.36 ± 0.03 ^de^
IVF	0.22 ± 0.02 ^b^	0.36 ± 0.04 ^b^	0.38 ± 0.03 ^b^	0.43 ± 0.01 ^cd^
SVFM	0.25 ± 0.04 ^ab^	0.34 ± 0.01 ^bc^	0.37 ± 0.01 ^b^	0.42 ± 0.09 ^cd^
IVFM	0.26 ± 0.04 ^ab^	0.35 ± 0.07 ^bc^	0.38 ± 0.06 ^b^	0.52 ± 0.02 ^bc^
SVFF	0.26 ± 0.02 ^ab^	0.36 ± 0.02 ^b^	0.46 ± 0.03 ^a^	0.58 ± 0.02 ^ab^
IVFF	0.33 ± 0.04 ^a^	0.45 ± 0.01 ^a^	0.53 ± 0.04 ^a^	0.68 ± 0.07 ^a^

C (control); SVF (sprayed vaccinated feed); IVF (incorporated vaccinated feed); SVFM (sprayed vaccinated feed with mineral oil); IVFM (incorporated vaccinated feed with mineral oil); SVFF (sprayed vaccinated feed with fish oil); IVFF (incorporated vaccinated feed with fish oil). Values are shown as mean ± SD (*n* = 2). Different lowercase letters within the same column indicate statistically significant differences (*p* < 0.05) based on post-hoc comparisons.

**Table 9:** Relative percentage survival rate of vaccinated and control groups.

Treatments	Total No. of Fish	No. of Dead	Protection (%)	Mortality (%)	RPS (%)
C	15	12	20	80	----
SVF	15	10	34	66	18
IVF	15	9	40	60	25
SVFM	15	8	47	53	34
IVFM	15	8	47	53	34
SVFF	15	5	67	33	58
IVFF	15	2	87	13	83

C (control); SVF (sprayed vaccinated feed); IVF (incorporated vaccinated feed); SVFM (sprayed vaccinated feed with mineral oil); IVFM (incorporated vaccinated feed with mineral oil); SVFF (sprayed vaccinated feed with fish oil); IVFF (incorporated vaccinated feed with fish oil); RPS (relative percentage survival).

## Data Availability

The original contributions presented in this study are included in the article. Further inquiries can be directed to the corresponding authors.
